# The Infection of Crime

**Published:** 1869-06-15

**Authors:** 


### *The Infection of Crime.*

It is a noticeable phenomenon in the moral world, not only that local epidemics of crime and suicide occur from time to time, but that a certain kind of crime, or mode of killing, assumes temporary prominence in these epidemics. The more fantastical in horror be an evil deed, the surer is it to be followed by imitative repetitions. Let us see whether there be a physiological explanation of this fact.

Without pushing the argument of "moral insanity" as far as do some "counsel for the defence," who seem to hold that the mere commission of an unlawful act is in itself evidence of mental aberration—without even quoting the concession made by law to the church, in attributing temporary insanity to every suicide, so that he may have a christian burial—we hazard little in asserting that for the perpetration of the graver forms of crime, there must exist a morbid pre-disposition which may be characterized at least as unsoundness of mind. The man who deliberately takes his own or another's life follows an unnatural purpose, and thus far shows an unsettled mind, although in legal definition he may be sane. He who surrounds a causeless crime with circumstances of grotesque atrocity, though he also may satisfy the technical meaning of sanity, and justly suffer the legal penalty of his act, is nevertheless undoubtedly diseased in mind, physiologically speaking. Those perversions of impulse which, when developed to actual insanity, we call Pyromania, Kleptomania, Homicidal or Suicidal mania, have their minor degrees, ordinarily under the control of reason and unsuspected in their latency, save, perhaps, as matters of subjective consciousness.

Now, we know that in perturbation of the mental functions the imitative faculty is apt to become unduly roused. We have all noticed the apparent contagiousness of hysterical convulsions in the female wards of hospitals; of epilepsy among patients subject to that disease; and past and present history teaches us, in the "possessions" of Loudon and Louviers, and in the various vagaries of religious and political fanaticism, that "moral convulsions," so to speak, are even more infectious in their nature. In either case a dormant pre-disposition is roused to action and emancipated from the control of the judgment by a suggestive example.

With regard to crime, this mischievous stimulous of example is held up to view by elaborately sensational reports in the public press. If some frenzied miscreant batter out the brains of his wife and children with a fence-rail, and afterwards burn their bodies, straightway some enterprising newspaper covers its first page with a florid description of all the disgusting details; the feast of horror is spread at every breakfast table throughout the country; and immediately fence-rails and incineration come into fashion, and domestic holocausts are the order of the day.

Or some original genius devises a new method of putting an end to himself, and at once the morning papers "Herald" to the "World" his achievement, with a similar result of calling out a host of imitators. Many a man may have a proclivity towards unprovoked homicide, or self-destruction, which would have remained latent were it not excited beyond restraint by the overdone sensational journalism of the day; nay, more, it may be safely said that no man free from such proclivity could relish the perusal of such details.

If there be reasonable grounds for what we have said we trust that the conductors of reputable journals will give it thought and be guided by a regard for the public welfare. For those vile weekly prints which exist only by pandering to the depraved appetite for horror of the worst classes of the community, mere counsel will not suffice, and we must await the time when their villainous incentives to crime will be recognized and suppressed by law.—*N. Y. Medical Gazette.*

Prolonged Anuria.—Cases of suppression of the urinary secretion, as in cholera, persisting for weeks and months, are not frequent, and their rarity equals their gravity.

A woman twenty-seven years of age, married but childless, after five months' suffering from amenorrhœa, and leucorrhœa, consulted Dr. Gallina, because she had not passed urine for twenty-four hours. Catheterism only yielded a few drops of coffee colored liquid, and for the suceeding eight days no more of it appeared. Application of leeches to the perineum, nitrate of urea internally, and warm baths were administered up to the twenty-fifth day. No effects having been produced, the patient consulted Dr. Albertini, at the Milan Hospital, who after two hours' minute exam ination, found absolutely no lesion to explain this failure of secretion, nor any alteration resulting from it. Her general health had suffered in no respect. Prof. Rudolph being called into consultation, considered the suppression was due to amenorrhœa. Emmenagogues were administered, and the menses appeared. At the same time, six hundred grammes of urine were extracted by catheterism on the forty-third day, and the normal secretion became established without the least injury to health.—*Gazette Medicale de Lombardia, and Western Journal of Medicine.*

---

The Physiological Action of Picrotoxin.—M. Rober has recently been engaged in experimenting on this substance, which was obtained as long ago as 1812, by M. Boullay from the well known seeds of the Cocculus Indicus. The composition of picrotoxin is represented by the formula $C_{20}H_{12}O_8$ and its reaction is neutral. The conclusion at which M. Roeber arrives from his investigations is that picrotoxin is a powerful exciter of the medulla oblongata, or, perhaps, to express it more accurately, of all the nerve-centres which are situated in the medulla oblongata. Hence follows violent and long continued convulsions of the body generally, prolongation and ultimate stoppage of the cardiac beats, and acceleration of the respiration, which is finally arrested by cramp of the glottis and of the diaphragm. Though the drug does not appear to be at the present available as a therapeutic agent, it is interesting as affording and example of the action of a poisonous substance on a particular section of the nervous system.—*Lancet.*

### *A Caution to Prescribers.*

[The following communication from Prof. Chas. A. Joy conveys a caution worthy of the serious attention of every practitioner.]

EDITORS MEDICAL JOURNAL:

There is great danger in prescribing oxidizing agents in the same compound with the Iodide of Bromide of Potassium.

Chlorate of potash and Iodide of Potassium dissolved together in water crystallize out separately, but these two salts taken into the stomach re-act upon each other, and the resulting Iodate of potash acts as a violent poison.

I presume that chlorate of potash and Bromide of potassium would behave in a similar way, and I should be afraid to mix caustic potash or any alkali with the Bromide of potassium. C. A. J.

---

### *Brainard Medical Society.*

Society met in Miramac, Indiana, June 2, 1869. Members present, Drs. F. B. Thomas, H. Kittinger, G. W. Reddich and P. H. Leavitt, Miramac; L. D. Glazebrook, San Pierre; J. B. Hoag, Knox; Wm. Perry, North Judson; Jas. Tolerton, Rochester; J. W. C. Eaton, Pulaski; W. T. Cleland, J. H. Smith, A. R. Thompson and R. W. Jackson, Kewana, and I. B. Washburn, Star City.

Officers elected at the paevious meeting were:

J. W. C. Eaton, President; I. B. Washburn, Secretary; J. H. Smith, Treasurer; F. B. Thomas, P. H. Leavitt and R. W. Jackson, Censers.

Dr. Eaton, before taking the chair, read an able and instructive address.

Dr. Hoag read an essay on "The Application of Consevative Medicine to Hygiene.

The Society was divided into five Sections. The members chose the sections to which they wished to belong, and elected the chairmen of the same as follows:

ANATOMY, PHISIOLOGY AND SURGERY.—Dr. F. B. Thomas, Chairman; Glazebrook, Tolerton, Hoag, Cleland and Leavitt.

TAEORY AND PRACTICE OF MEDICINE.—Dr. L. D. Glazebrook, Chairman Washburn, Smith, Perry, Leavitt and Thomas.

OBSTETRICS AND DISEASES OF WOMEN AND CHILDREN.—Dr. T. B. Washburn, Chairman; Tolerton, Hoag, Thompson, Smith, Thomas and Glazebrook.

EYE AND EAR.—Dr. J. B. More, Chairman; Glazebrook and Thomas.

Drs. Hoag, Thomas and Glazebrook were appointed a Committee on Resolutions on the death of Dr. A. M. Pearson, of Miramac.

Adjourned to meet in Miramac, Indiana, July, 7, 1869.

I. B. WASHBURN, M.D., Sec'y.

